# Decontamination of Surgical Instruments for Safe Wound Care Surgeries in Disasters: What are the Options? A Scoping Review

**DOI:** 10.1017/S1049023X2100090X

**Published:** 2021-10

**Authors:** Anna Rowinski, Johan von Schreeb

**Affiliations:** Department of Global Public Health, Karolinska Institutet, Stockholm, Sweden

**Keywords:** decontamination, disasters, disinfection, sterilization, surgical instruments

## Abstract

International guidelines stipulate that autoclavation is necessary to sterilize surgical equipment. World Health Organization (WHO) guidelines for decontamination of medical devices require four levels of decontamination: cleaning, low- and high-level disinfection, as well as sterilization. Following disasters, there is a substantial need for wound care surgery. This requires prompt availability of a significant volume of instruments that are adequately decontaminated. Ideally, they should be sterilized using an autoclave, but due to the resource-limited field context, this may be impossible. The aim of this study was therefore to identify whether there are portable and less resource-demanding techniques to decontaminate surgical instruments for safe wound care surgery in disasters. A scoping review was chosen, and searches were performed in three scientific databases, grey literature, and included data from organizations and journals. Articles were scanned for decontamination techniques feasible for use in the resource-scarce disaster setting given that: they achieved at least high-level disinfected instruments, were portable, and did not require electricity. A total of 401 articles were reviewed, yielding 13 articles for inclusion. The study identified three techniques: pressure cooking, boiling, and liquid chemical immersion, all achieving either sterilized or high-level disinfected instruments. It was concluded that besides autoclaves, there are less resource-demanding decontamination techniques available for safe wound surgery in disasters. This study provides systematic information to guide optimal standard setting for sterilization of surgical material in resource-limited disaster settings.

## Introduction

Disasters are events that kill, maim, and create needs that exceed available health care capacities. Natural disasters such as typhoon and flooding cause a significant burden of minor injuries requiring surgical wound care.^1–3^ This requires surgical instruments that must be decontaminated to avoid transmission of pathogens.^4,5^ There are different degrees of decontamination: clean, disinfected, and sterile.^5,6^ *Disinfected* means that microorganisms have been destroyed or removed but does not necessarily include destruction of bacterial spores. *Sterilization* is the process used to render an object free from viable microorganisms, including viruses and bacterial spores, but not prions.^6,7^ The Spaulding classification is a widely accepted classification for decontamination of medical devices.^5^ Surgical instruments and other reusable medical devices are classified into Critical, Semi-Critical, and Non-Critical devices based on their risk to spread infections.^5,7^ Sterilized instruments (critical devices) are mandatory for surgery on intact skin while high-level disinfected instruments (semi-critical devices) are considered sufficient for non-intact skin, such as open wounds.^4,7^ Common microbes found in open wounds are eradicated when instruments are disinfected.^7,8^ However, spores from a few spore-forming bacteria may survive disinfection and potentially cause infections. However, it remains unclear to what extent this has clinical value in the disaster setting where wounds already are contaminated.

Following the chaos of unregulated international health care assistance after the 2010 Haiti earthquake, the World Health Organization (WHO; Geneva, Switzerland) and partners developed standards and classification for Emergency Medical Teams (EMTs) deployed to natural disasters.^9^ In these, EMT Type 1 provides fixed or mobile out-patient care including basic wound care.^9^ The EMT standards stipulate that wound care should only be done with disposable or autoclaved instruments using high pressure and steam.^9^ However, EMT Type 1 mobile experiences from the 2015 earthquake in Nepal, as well as the 2019 Mozambique typhoon, have highlighted significant logistical problems (ie, weight, size, and lack of electricity) with bringing an autoclave or sufficient disposable instruments to remotely affected areas to manage a significant number of minor wounds. To ensure rapid and mobile health response to disasters, there is need for decontamination techniques that are safe but also easy to both deploy and use in a resource-scarce field setting. This article aims to assess portable and non-electricity-dependent alternative techniques for safe decontamination of surgical material for minor wound surgery in disaster settings.

## Materials and Methods

This scoping review followed established scoping review methodology defined by Arksey and O’Malley.^10^ The Preferred Reporting Items for Systematic Reviews and Meta-Analyses Extension for Scoping Reviews (PRISMA-ScR) protocol^11^ was used. Three databases were searched (PubMed [National Center for Biotechnology Information, National Institutes of Health; Bethesda, Maryland USA], Web of Science [Thomson Reuters; New York, New York USA], and Global Health [EBSCO Information Services; Ipswich, Massachusetts USA]); grey literature via additional databases (Global Index Medicus [WHO; Geneva, Switzerland], Popline [Johns Hopkins Bloomberg School of Public Health; Baltimore, Maryland USA], Grey Matters [CADTH; Ottawa, Ontario, Canada], BASE, WHO HQ, JBI [Ovid Technologies; New York, New York USA], and INAHTA [Institute of Health Economics; Edmonton, Alberta, Canada]); data from relevant organizations (WHO, Médecins Sans Frontières [MSF; Geneva, Switzerland], and the International Committee of the Red Cross [ICRC; Geneva, Switzerland]); and various journals.

Articles were scanned for safe and feasible decontamination techniques. A technique was considered feasible if it was portable or did not require a power grid. Portability in this study is defined using the International Air Transport Association (IATA; Montreal, Quebec, Canada) recommended weight limit, stating that the maximum weight of any single piece should not exceed 23kgs.^12^ Minor wound surgery was defined as surgical management of already open and contaminated minor wounds that can be managed at a primary health service level such as EMT Type 1. A technique was considered safe if it achieved semi-critical decontamination level by either sterilization or high-level disinfection. A thematic analysis of the findings was performed and a numerical summary of the searches outlined using a PRISMA flowchart.^11,13^ The results were compiled in two tables.

**Table 1.** An Introduction to the Techniques

	Technique	Description	Mechanism of Action	Readily Available
**Techniques Achieving Sterilization**				
Portable Sterilization	Portable Nitrogen Dioxide Sterilizer^14^	A polypropylene case, in the size of a briefcase.	Non-steam autoclave using nitrogen dioxide gas.	No
	Portable Chlorine Dioxide Sterilizer^15,16^	A plastic case, in the size of a briefcase. Developed by Natick Soldier Center.	Non-steam autoclave using chlorine dioxide gas.	No
	ROSS M1 Portable Ozone Sterilizer^17^	A plastic case in the size of a briefcase. Developed for and evaluated by US Special Forces.	Non-steam autoclave using peroxone gas.	No
Pressure Cooking	Specially Designed Low-Cost Autoclave^18^	Developed at MIT for and used by health care centers in Nepal and marketed under the brand OttoClave.^19^	Design included a heating element, pressure cooker, pressure sensor, and monitor.	Unknown
	Pressure Cookers^20,21^	Five commercially available pressure cookers tested on kerosene and gas stoves.	Steam under pressure.	Yes
	Portable Steam Sterilizer^22^	Originally designed by WHO and now adapted for use in sterilizing intrauterine device insertion instruments.	Steam under pressure.	Unknown
Liquid Chemical Immersion	Liquid Chemicals^23^	A number of liquid chemicals available, achieving sterility if soaked for up to 12 hours. The most common is glutaraldehyde.	Inactivates microbes if instruments are properly cleaned.^23^	Yes
Solar Driven	Solar Driven Dry Heat Oven^24^	Maria Telke’s solar oven; can generate temperatures above 180°C.	Dry heat.	Unknown
	Broadband Light-Absorbing Nanoparticles as Solar Photothermal Heaters^25^	As light is absorbed by a nanoparticle, a temperature difference is established which converts the liquid into vapor. When the vapor reaches the surface, it is released, resulting in steam generation at lower temperatures than the boiling point.	High-temperature steam.	No
UVC Light	Chlorhexidine Scrub + UVC Light^26^	Each instrument exposed to UVC light radiation by waving the wand up and down the side of the instrument for 45 seconds.	UVC light disrupts the DNA of microorganisms.	No
**Techniques Achieving High-Level Disinfection**				
Boiling	Boiling^27^	According to WHO guidelines on sterilization and disinfection methods effective against HIV (1989), if sterilization is not possible, high-level disinfection by boiling for 20 minutes is acceptable.^27^	High temperature.	No Specific Appliance Required
Liquid Chemical Immersion	Liquid Chemicals^23^	A number of liquid chemicals are available, achieving high-level disinfection if soaked for 15-30 minutes. The most common is glutaraldehyde.	Inactivates microbes if instruments are properly cleaned.^23^	Yes

Abbreviation: UVC light, ultraviolet C light

**Table 2.** Techniques that are Portable and do not Require a Power Grid

	Technique	Portability	Requires a Power Grid	Chamber Volume	Cycle Time
**Techniques Achieving Sterilization**					
Portable Sterilization	Portable Nitrogen Dioxide Sterilizer^14^	Yes	No	9L	3-8 hours
	Portable Chlorine Dioxide Sterilizer^15,16^	Yes	No	n/a	30 minutes
	ROSS M1 Portable Ozone Sterilizer^17^	Yes	No	18L	45 minutes
Pressure Cooking	Specially Designed Low-Cost Autoclave^18^	Yes	No	n/a	30 minutes
	Local Market Pressure Cooker^20^	Yes	No	12L	30-40 minutes
	Four Local Market Pressure Cookers^21^	Yes	No	8L	15 minutes/ 16 psi 40 minutes/ 13 psi
	Portable Steam Sterilizer^22^	Yes	No	8L	20 minutes
Liquid Chemical Immersion	Liquid Chemicals^23^	Yes	No	n/a	Up to 12 hours
Solar Driven	Solar Driven Dry Heat Oven^24^	Yes	No	n/a	40 minutes at 180°
	Broadband Light-Absorbing Nanoparticles as Solar Photothermal Heaters^25^	Yes	No	14.2L	30 minutes
UVC Light	Chlorhexidine Scrub + UVC Light^26^	Yes	No	n/a	45 seconds
**Techniques Achieving High-Level Disinfection**					
Boiling	Boiling^27^	Yes	No	n/a	20 minutes
Liquid Chemical Immersion	Liquid Chemicals^23^	Yes	No	n/a	15-30 minutes

Note: Not applicable (n/a) - the information could not be found.

Abbreviations: PSI, pound-force per square inch (unit of pressure); UVC light, ultraviolet C light.

## Results

**Figure 1.**
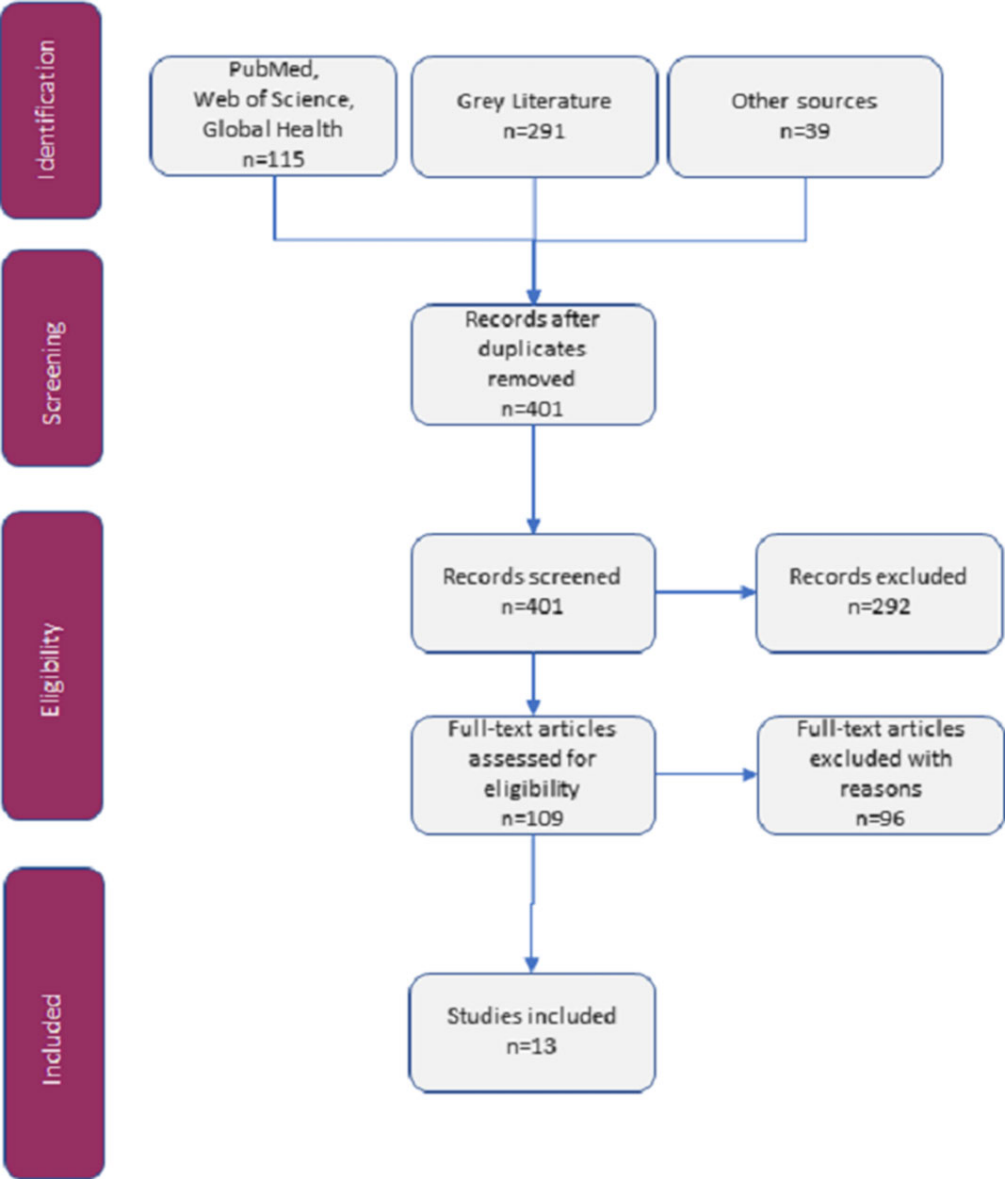
PRISMA-ScR Flowchart: Search Results. Abbreviation: PRISMA-ScR, Preferred Reporting Items for Systematic Reviews and Meta-Analyses Extension for Scoping Reviews.

The search rendered 401 articles that were screened for eligibility (Figure 1). Following screening, a total of 13 records matched the inclusion criteria. The 13 studies described six techniques, out of which three were easily available for use or purchase. The identified techniques achieving either sterilized or high-level disinfected instruments were pressure cooking, boiling, and liquid chemical immersion. They are all portable techniques and do not require a power grid.

The feasible and safe techniques identified in this study are presented in two tables and are shown as either achieving sterilized or high-level disinfected instruments. Table 1^14-27^ contains a brief introduction of the techniques. The attributes of the techniques are presented in Table 2: portability, chamber volume, cycle time, and whether it requires a power grid or not.

## Discussion

This scoping study found 13 articles presenting six techniques that can sufficiently decontaminate instruments for safe minor wound surgery and seem feasible to use in disaster settings. The identified techniques are easier to use and transport to remotely affected disaster areas compared to heavy and electricity-dependent autoclaves. It was found that safe alternatives to more resource-demanding autoclaves that are currently defined as minimum standards for EMT Type 1 are available.^9^ Pressure cooking, liquid chemicals, and boiling are the techniques that can easily be available for purchase or use today. Pressure cooking or boiling are the most favorable techniques, considering the potential toxicity and residue of liquid chemicals.^5,6^ In addition, the WHO does not recommend liquid chemicals for sterilization. It is difficult to control the process and there is a risk of contamination during the rinse of residual chemicals before patient use.^4,6^ Pressure cooking for 15-40 minutes achieves a higher level of decontamination than boiling for 20 minutes. A pressure cooker can be purchased on local markets while boiling is even easier to achieve with limited resources. In addition, pressure cooking and boiling are more sustainable than disposables and do not require transportation of large quantities of disposables in order to perform the number of wound surgeries often needed after a disaster.

According to Spaulding, high-level disinfected instruments are acceptable for instruments in contact with non-intact skin,^4,5,7^ but what are the safety implications of using high-level disinfected instead of sterilized instruments in a disaster setting? A systematic review focusing on the nature of wounds in disaster situations studied the most common organisms of infected wounds.^28^ All of those pathogens are removed with high-level disinfection.^5^ However, it remains undocumented to what extent high-level disinfected instruments could pose risks in the disaster setting as spores are not fully eliminated. Spores of concern in the disaster setting are Clostridium perfringens and tetani. Tetanus cases have been reported following natural disasters in areas with low tetanus vaccination coverage.^29^ While non-sterile instruments theoretically could spread tetanus, this has not been documented in practice. Poor early wound care is often complicated by more extensive infection and tissue necrosis, gangrene, sepsis, and mortality.^30^ Wound infections and complications including tetanus may develop if surgical cleaning and debridement is not adequately carried out. Spore transmission and infection could besides debridement be avoided (but not replaced) by preventive antibiotic regimes.^28^ With this in mind, one should aim to autoclave instruments, but when this is not feasible, pressure cooking is an acceptable alternative if combined with tetanus prophylaxis.

## Methodological Considerations/Limitations

The scoping review methodology enables searching both published and unpublished material, suitable in this area where important evidence might not yet have been published. Nonetheless, using such a broad search scope makes is challenging to establish boundaries. Therefore, a well-defined search strategy was developed. For this project, other methods could have been relevant to use, for example, a qualitative interview-based method. However, this would impose the risk of only increasing knowledge about current processes, not finding research on new developments. Another example is a systematic review which might have yielded a larger number of articles, yet carrying the risk of missing important evidence from unpublished sources. It would also have been interesting to compare these results with similar research, but no previous research in a similar area was found. In summary, a scoping study is particularly relevant to disciplines with emerging evidence such as this study.

## Conclusion

This study found alternative safe, easily deployable, and feasible techniques to decontaminate surgical instruments for wound management in disaster settings, such as pressure cookers, boiling, and liquid chemicals. The study result suggests that current EMT Type 1 decontamination standards for minor wound surgery instruments could be adapted.
